# Effects of inoculants on the quality of alfalfa silage

**DOI:** 10.3389/fmicb.2025.1541454

**Published:** 2025-05-12

**Authors:** Si-Yi Wang, Yuan-Yuan Jing, Guolin Yang, Bin Liu, Feng-Qin Gao

**Affiliations:** ^1^Institute of Grassland Research Chinese, Academy of Agricultural Science, Hohhot, China; ^2^College of Grassland Science, Qingdao Agricultural University, Qingdao, China; ^3^Animal Husbandry Institute of Inner Mongolia Academy of Agricultural and Animal Husbandry Sciences, Hohhot, China

**Keywords:** WL298HQ alfalfa, silage agent, fermentation quality, nutritional quality, membership function

## Abstract

To explore the effects of different silage inoculants on the silage quality of alfalfa (*Medicago sativa* L.), the experiment used Alfalfa with a moisture content of 60.00% after harvesting as the raw material. The treatments included a control group containing only distilled water (CK), Xinlaiwang I-straw silage agent (A), Xinlaiwang I-alfalfa silage agent (B), Zhuanglemei silage fermentation agent (C), Baoshiqing (D), and Kangfuqing S lactic acid bacteria inoculant (E), totaling six treatments. After 60 days of normal temperature sealing treatment, the silage fermentation and nutritional indicators of Alfalfa were measured, and the silage fermentation effect was analyzed by the membership function method. The experiment showed that when the moisture content of alfalfa was 60.00%, the neutral detergent fiber (NDF) and acid detergent fiber (ADF) contents of the silage agent treatment groups were significantly lower than those of the CK group (*p <* 0.05). The lactic acid (LA) content was significantly higher in the treatment group than in the CK group (*p* < 0.05). The addition of Xinlaiwang I-alfalfa silage agent in group B significantly increased the crude protein (CP) and LA levels in the Alfalfa silage (*p* < 0.05). It also reduced the neutral detergent fiber (NDF) and acid detergent fiber (ADF) contents. Additionally, the pH and Ammonia Nitrogen/Total Nitrogen (AN/TN) ratio were lowered. Propionic acid (PA) and butyric acid (BA) were not detected. After the membership function calculation, the average membership value of Xinlaiwang I-alfalfa silage agent (B) group was the highest with a score of 0.90, ranking first, and the silage quality was the best. In summary, through quality analysis and membership function calculation, Xinlaiwang I-alfalfa silage agent can effectively improve the silage quality of WL298HQ alfalfa.

## Introduction

In recent years, the global shortage of high-quality forage has led to a persistent imbalance between grass and livestock, severely affecting the development of animal husbandry. To alleviate this acute contradiction, the United States Department of Agriculture (USDA) provides subsidies and supports research to promote the development of alfalfa industries. The European Union’s Common Agricultural Policy (CAP) includes direct payments and support measures for the grass industry, encouraging sustainable grass production and ensuring the supply of forage for grass-based livestock. The Australian government promotes the development of the alfalfa industry through research and development support and market access assistance. In 2023, the Chinese Central No. 1 Document was released. It clearly accelerates the development of the grass industry. This includes the promotion of alfalfa. The document implements several policies. These policies aim to increase grass production. They also focus on saving grain. Another goal is to convert grain into forage. Additionally, the document aims to revitalize the dairy industry through the development of alfalfa. These measures are designed to improve the agricultural production structure. They focus on building high-quality, water-saving, high-yield, and stable feed production bases (Zhong, 2024). The goal is to advance the alfalfa and other forage industries under the “big food concept” for a diverse food supply system. The plan involves using land resources like saline-alkali, dry, and sandy lands efficiently. It promotes planting high-yield, high-protein, high-quality forages like alfalfa, corn silage, and oats. This approach aims to develop both grain and forage resources to satisfy the growing dietary diversity and consumption needs of the global population (Zhu et al., 2024).

Alfalfa (*Medicago sativa* L.) has characteristics such as salt tolerance, resistance to poor soil, and nitrogen fixation. The main processing methods of alfalfa globally include grass pellets, blocks, bundles, and powder, which have relatively low efficiency and added value. These methods also increase the risk of rain damage and leaf loss (Ye, 2018). Alfalfa silage perfectly avoids these risks, ensuring a stable supply of high-quality forage for dairy and beef production globally (Muck et al., 2018). To maximize the retention of nutritional components in alfalfa forage, it is important to improve quality and efficiency. Optimizing alfalfa silage methods is crucial for this purpose. Enhancing silage efficiency (Wang Y. et al., 2023) is vital, which will directly impacts the improvement of animal production performance and product yield. Silage is a process carried out under anaerobic conditions, where lactic acid bacteria (LAB) degrade water-soluble carbohydrates (WSC) to produce lactic acid (LA), lowering the pH of the silage and inhibiting microbial activity to preserve it for a long time. Silage methods include conventional silage, semi-dry silage, and inoculant silage. The microbial community in silage raw materials includes beneficial microorganisms such as LAB, as well as potential spoilage microorganisms such as molds and yeasts. Changes in the abundance and composition of surface-adhered bacteria during forage harvest are crucial for the natural fermentation process of silage and the subsequent development and succession of the microbial community. Therefore, improving silage processing and production is extremely important for the development of animal husbandry.

Adding lactic acid bacteria can enhance silage quality and stability. Wang X.-C. et al. (2024) discovered that various treatments, including bacterial agents, enzyme preparations, their combinations, propionic acid, and formic acid, increase crude protein in oat and alfalfa silage. Mejía-Avellaneda et al. (2022) showed that the use of lactic acid bacteria (LAB) inoculants for silage has become a major alternative method to improve milk and meat production in cattle. The production of lactic acid bacteria inoculants involves microbiological and technological aspects, including biomass acquisition, co-culture, probiotic addition, antimicrobial peptide production, bioreactor operation, and the formulation of the final product. Diogénes et al. (2023) demonstrated that the use of inoculants in silage has great application prospects. Adding *Lactobacillus buchneri* as a fermentation inoculant significantly enhances silage quality by improving fermentation, lowering mycotoxin levels, increasing aerobic stability, and boosting ruminant cell wall digestibility and nitrogen use efficiency. Niderkorn and Jayanegara (2021) found that adding legumes containing Phytochemicals and PBC to silage can provide multiple and cumulative effects on silage fermentation and silage product quality. Menezes et al. (2022) grouped the data into subgroups according to the type of inoculant (Mho = homologous fermentation microbial inoculant; Mhe = heterologous fermentation microbial inoculant; MMi = microbial inoculant mixture; Fa = formic acid; En = enzyme). Using the “meta” package in R software for meta-analysis, all inoculants enhanced silage quality, with MMi boosting sheep weight gain and Mhe improving aerobic stability, offering insights for commercial farms. Wang Y.-P. et al. (2024) showed that substituting part of the peanut seedling diet with silage from alfalfa, corn, or mulberry enhanced sheep growth, slaughter performance, and immunity, with alfalfa silage yielding the best results. Optimizing silage techniques is crucial for diversifying roughage sources, especially during feed shortages.

The study used Xinlaiwang silage inoculants and others on alfalfa silage to assess their impact on quality, summarizing the selection and use of inoculants to theoretically support finding the optimal microbial flora for alfalfa silage.

## Materials

### Materials

The target variety of this experiment was WL298HQ alfalfa with high cold resistance planted in the base of the Academy of Agricultural Sciences in Bayannur, Inner Mongolia Autonomous Region. It was harvested at the initial flowering stage of the third cutting, with stubble height controlled between 8 to 10 cm, at which time the moisture content of alfalfa was approximately 75%. It was dried to a moisture content of about 60%.

As shown in Table 1, the dry matter (DM) and crude protein (CP) content of the Alfalfa raw material were 40.00% and 21.08%, respectively, with water-soluble carbohydrates (WSC) accounting for 2.55% of the total nutritional components. The inoculant is shown in Table 2.

**Table 1:** Raw materials nutrition content composition of WL298HQ alfalfa.

DM, %FM	CP, %DM	NDF, %DM	ADF, %DM	WSC, %DM	RFV, %
40.00	21.08	37.03	29.19	5.55	183

FM, Fresh alfalfa; DM, Dry matter; FM, Fresh matter; CP, Crude protein; NDF, Neutral detergent fiber, ADF, Acid detergent fiber; WSC, Water soluble carbohydrate. RFV, Relative Feed Value; The same below.

**Table 2:** Source, composition and content of silage agent.

Microbial colony/(CFU·g^−1^) and other components	Xinlaiwang I-straw silage agent	Xinlaiwang I-alfalfa silage agent	Zhuanglemei silage fermentation agent	Baoshi silage	Kangfuqing S Lactic acid bacteria silage agent
*L. plantarum*	≥1 × 10^10^	≥1 × 10^11^	≥1.6 × 10^9^	≥2.5 × 10^10^	ND
*P. pentosaceus*	≥1 × 10^10^	ND	ND	≥2.5 × 10^10^	ND
*L. buchneri*	≥1 × 10^10^	ND	≥4.0 × 10^8^	≥2.5 × 10^10^	≥1 × 10^11^
*L. casei*	ND	≥1 × 10^11^	ND	ND	ND
Water content	ND	ND	<10.0%	ND	ND
Source	Xinlaiwang Biotechnology Co., Ltd	Xinlaiwang Biotechnology Co., Ltd	Sichuan Gaofu Ji Biotechnology Co., LTD	Newman Agricultural Trading Shanghai Co., LTD	Aikang Dalian environmental protection Products Co., LTD

ND means not detected. *L. plantarum, Lactobacillus plantarum; P. pentosaceus, Pediococcus pentosaceus; L. buchneri, Lactobacillus buchneri; L. casei, Lactobacillus casei*; CFU, colony-forming units. The same below.

## Methods

### Experimental design

The experiment included six treatments. Adding silage inoculant Xinlaiwang I-silage agent for straw (A), Xinlaiwang I-silage agent for alfalfa (B), Zhuanglemei silage fermentation (C), Baoshiqing (D), Kangfuqin S lactic acid bacteria silage agent (E). The last group is the control group without any inoculants (CK). The specifics of these groups are provided in Table 3. The harvested alfalfa was dried to about 60% moisture content, cut into 1 or 2 cm segments, thoroughly mixed with the inoculants, and then placed in vacuum silage bags (200 × 250mm). Approximately 300 grams of each treatment were sampled, sealed using a vacuum machine, and stored at room temperature. There were a total of 18 treatments, each replicated three times. After 60 days of fermentation, the silos were opened, and the fermentation quality and nutritional components were analyzed.

**Table 3:** Experimental design.

Material	Treatments					
WL298HQ (Alfalfa)	Distilled water (CK)	Xinlaiwang I-straw silage agent (A)	Xinlaiwang I-alfalfa silage agent (B)	Zhuanglemei silage fermentation agent (C)	Baoshiqing silage (D)	Kangfuqing S Lactic Acid bacteria silage agent (E)

### Determination indicators and methods

After 60 days of silage, 25 g of the silage were taken, added to 180 mL of sterile injection water, and mixed evenly. The mixture was then placed in a juicer and run for 1 min, followed by filtration through four layers of gauze and qualitative filter paper. The mixture was placed in a 4°C refrigerator and left to stand for 24 h to detect the fermentation indicators of the final alfalfa mixture extract. Lactic acid (LA) content was measured using standard (Zeng and Cao, 2017) DB15/T1458-2018 (Xu et al., 2017). Ammonia nitrogen (AN) and its ratio to total nitrogen (AN/TN), acetic acid (AA), propionic acid (PA), and butyrate acid (BA) levels were also determined by this standard. The calculation formula is: total nitrogen content = crude protein ÷ 6.25. The fermentation quality of the alfalfa silage was evaluated using the V-score scoring system (Gao et al., 2020). One hundred grams of Alfalfa silage samples were dried at 65°C for 48 h before nutritional quality determination. DM was determined using the drying method (Liu et al., 2024), according to the recommended national standard GB/T6435-2014. Crude protein (CP) content was determined according to the recommended national standard GB/T6432-2018 (Xu et al., 2021). Crude ash (Ash) content was determined according to the recommended national standard GB/T6438-2007 (National Feed Industry Standardization Technical Committee, 2006; National Feed Industry Standardization Technical Committee, 2014; National Feed Industry Standardization Technical Committee, 2018; Zhang, 2003). Neutral detergent fiber (NDF) and acid detergent fiber (ADF) (Li et al., 2021; Van Soest et al., 1991) were determined according to the recommended national standard GB/T20806-2006. Water-soluble carbohydrates (WSC) were determined according to the method of Xu et al. (2017). The pH of the filtrate was measured using a LAQUAtwin-pH-22 (Inner Mongolia Autonomous Region Animal Husbandry Standardization Technical Committee, 2018) portable precision pH meter (Wu et al., 2006).

### Data statistics and analysis

Excel 2010 and SPSS 26.0 software were used to create tables and analyze the data. One-way ANOVA was performed to examine the differences between treatments, followed by multiple comparisons using Duncan’s method. The results were presented as “mean ± standard error,” and a *p* < 0.05 was considered to indicate a significant difference.

## Results and discussion

### Effects of different silage inoculants on the nutritional quality of alfalfa silage

The dry matter DM content of silage is a key factor affecting the fermentation process. Table 4 shows that the DM content in Group B was significantly higher than in Groups A, C, and E (*p* < 0.05), indicating good fermentation control and less nutritional loss in Group B. A higher DM content in silage can have a negative impact on the activity of plant enzymes that hydrolyze complex water-soluble carbohydrates (WSC), leading to DM loss rates as high as 4%~20% (Zhao et al., 2022). In this experiment, the DM content of group B was increased after silage, which may be attributed to the bias of the sampling part to the bottom of the silage bag, similar to previous studies (Yang et al., 2015). DM, NDF, and ADF contents decreased with sampling depth in the silage container, while relative feeding value increased. Group B had significantly higher CP content than other groups (*p* < 0.05), likely due to the high concentration of *Lactobacillus casei* and *Lactobacillus plantarum* inhibiting protein decomposition enzymes, thus reducing protein breakdown in the silage. The protein retention rate in the silage improved due to the inoculants in group B. *Lactobacillus casei* also adjusted the microbial community structure. High moisture can cause dry matter loss in silage, but inoculants helped control moisture, reducing this loss (Han et al., 2022). Even with 60% water content in alfalfa, creating a high-water silage environment, group B’s inoculants maintained higher dry matter content, indicating effective reduction of dry matter loss in high-water conditions.

**Table 4:** Effects of different *Lactobacillus* treatments on nutrient quality of alfalfa silage.

Item CK		A	B	C	D	E
DM(%FM)	41.00 ± 1.00ab	39.67 ± 2.08b	44.67 ± 1.53a	39.00 ± 2.65b	41.33 ± 3.06ab	39.67 ± 3.21b
CP(%DM)	22.73 ± 0.67b	23.47 ± 1.12b	25.37 ± 0.06a	23.33 ± 1.40b	23.63 ± 0.31b	22.63 ± 0.75b
NDF(%DM)	33.85 ± 1.11ab	31.24 ± 1.32cd	29.96 ± 0.50d	31.33 ± 0.84cd	31.90 ± 1.09bc	33.28 ± 1.14ab
ADF(%DM)	26.61 ± 0.51ab	24.47 ± 0.68cd	23.83 ± 0.63d	25.26 ± 0.26bc	25.25 ± 0.59bc	26.22 ± 0.77ab
WSC(%DM)	1.66 ± 0.03ab	1.62 ± 0.08ab	1.83 ± 0.06a	1.59 ± 0.24b	1.55 ± 0.06b	1.74 ± 0.07ab
RFV(%DM)	149.63 ± 9.97ab	145 ± 22.52ab	166.67 ± 11.02a	152.67 ± 25.11ab	144.67 ± 10.02ab	133.33 ± 12.22b

DM, Dry matter; FM, Fresh matter; CP, Crude protein; NDF, Neutral detergent fiber, ADF, Acid detergent fiber; WSC, Water soluble carbohydrate. RFV, Relative Feed Value; The same below. The uppercase letters “A”, “B”, “C”, “D”, “E” and “CK” have been explained as per Table 3. Please refer to the explanations provided in the table. Lowercase letters, “a”, “b”, “c”, etc, indicate significant differences between data. Specifically, data with the same letters in the same column indicate no statistically significant difference, while data with different letters indicate significant differences.

It is speculated that group B inoculants could have several effects: they may contain *Lactobacillus plantarum* and *Lactobacillus casei*, which boost lactic acid bacteria growth (Wei et al., 2022). This could make lactic acid bacteria the dominant group, inhibiting aerobic bacteria and mold growth. Inhibition by *Lactobacillus casei* prevents silage spoilage and maintains feed quality. It retains nutrients like crude protein and amino acids by lowering pH and suppressing harmful microbes, enhancing the feed’s nutritional value. *Lactobacillus casei* addition reduces nutrient loss in silage and improves feed palatability and flavor (Xu et al., 2021). Even in a higher moisture environment, the pH can be rapidly reduced, the growth of harmful microorganisms can be inhibited, and the dry matter loss can be reduced. Similar to the results of previous studies, the water content of raw materials and the types of inoculants have positive effects on the quality of alfalfa silage. *Lactobacillus casei* can also degrade the anti-nutrient factors of silage and improve the utilization efficiency of feed (Yang et al., 2024). Even in a higher moisture environment, the pH can be rapidly reduced, the growth of harmful microorganisms can be inhibited, and the dry matter loss can be reduced. Similar to the results of previous studies, the water content of raw materials and the types of inoculants have positive effects on the quality of alfalfa silage (Yan et al., 2023). Inoculants may have altered the feed’s structure to decrease juice loss and preserve dry matter. Group B’s inoculants likely supplied extra nutrients like soluble carbs, offering a robust substrate for lactic acid bacteria to enhance fermentation. Netthisinghe et al. (2021) showed that CP and minerals (K, Ca, Mg) in feed, through the formation of AN and enhancement of feed buffering capacity, have an adverse effect on silage quality.

In this experiment, the high CP content of alfalfa, along with its strong buffering capacity and lack of WSC available for LAB fermentation, poses challenges for producing high-quality silage using conventional methods. Therefore, the addition of lactic acid bacteria preparations is an important way to promote lactic acid fermentation and improve silage quality. Previous studies indicate that adding lactic acid bacteria, glucose, and cellulase together: Increases DM, CP, and lactic acid content. Boosts the number of lactic acid bacteria. Decreases NDF and ADF content. Reduces the number of aerobic bacteria and molds. Enhances aerobic stability and rumen degradation of mixed silage. In this experiment, the inoculants in Group B significantly increased the CP content after silage compared to the control group, improving the quality of alfalfa silage, which is consistent with previous experimental results (Ma et al., 2023). Tian et al. (2022) found that using calcium propionate and a compound of lactic acid bacteria with calcium propionate (PACA) in silage significantly raised starch and soluble carbohydrate levels. This treatment also improved aerobic stability. Additionally, it simultaneously lowered the content of Aflatoxin B1 and yeast (*p* < 0.05). The combined addition of compound lactic acid bacteria and calcium propionate plays an important role in improving the quality and safety of silage.

The use of *L. buchneri* to produce coumarate esterase has achieved some success in improving silage digestibility. In addition, future silage inoculants are expected to directly inhibit clostridia and other harmful microorganisms, reduce high mycotoxin levels in post-harvest feed, enhance aerobic stability, increase cell wall digestibility, and improve the efficiency of nitrogen utilization by ruminants from silage, as well as increase the availability of starch.

The WSC content in Group B was significantly higher than in Groups C and D (*p* < 0.05). It is speculated that the reason for this result is that lactic acid and other antibacterial substances, such as bacteriocins, produced by *Lactobacillus plantarum* group B and *Lactobacillus casei* during the fermentation process inhibit the growth of other harmful microorganisms in the silage, thereby protecting WSC from consumption by these microorganisms. Similar to the study (Gao et al., 2020), its potent antibacterial properties may indirectly protect water-soluble carbohydrates (WSC) by suppressing microbial activity.

Zhang J. et al. (2022) found that a relatively low WSC level can lead to more nutrient loss, with WSC level being negatively correlated with aerobic stability. Wang H. R. et al. (2023) showed that *Bacillus coagulans* (BC) improved the fermentation quality of alfalfa silage, with the optimal combination being *Lactobacillus plantarum* (LP) and BC. According to the research results, BC can be considered a feasible biological resource for improving fermentation quality. Group B has the highest WSC and LA content. Despite WSC typically converting to lactic acid in silage, its elevation in Group B could be due to *Lactobacillus plantarum*’s production of bacteriocins and antimicrobial peptides that suppress harmful microbes. *Lactobacillus brucella*’s fermentation yields lactic acid and other acids, which lower pH and inhibit aerobic bacteria and yeast.

Koç et al. (2021) showed that adding kefir to alfalfa silage under low WSC conditions can improve fermentation quality and aerobic stability. Gümüş (2021) demonstrated that adding sucrose at different stages of silage has a positive impact on the nutritional value, fermentation characteristics, and relative feeding value of alfalfa silage. The WSC in Alfalfa was low, at 5.55% of its nutrients, possibly inadequate for complete lactic acid bacteria fermentation. Supplementing with sucrose in the experiment supplied extra fermentation substrates, fostering lactic acid bacteria growth and lactic acid production. Ren et al. (2024) found that DM and CP in silage decline during aerobic exposure (*p* < 0.05), suggesting Group B’s quality may result from inhibiting Gram-negative bacteria and clostridia, reducing fat oxidation. High-quality silage in Group B is attributed to effective sealing and compaction, limiting oxygen and microorganisms. Silage quality relies on forage choice and handling to maintain low pH and anaerobic conditions, inhibiting spoilage. Wilting and inoculants can address issues like low nitrate and buffering capacity (Macêdo et al., 2021). Single-strain lactic acid bacteria lack ideal silage-quality traits, necessitating mixed inoculant research (Han et al., 2022). Tannins and *Lactobacillus plantarum* can mitigate DM degradation inhibition, while their combination with *Bacillus subtilis* surpasses *Lactobacillus plantarum* alone in inhibiting aerobic spoilage. Sanchez et al. (2022) showed that adding wheat bran increased the potential and effective degradation rates of DM, CP, and NDF in silage, which is a characteristic of high-nutritional-quality silage. Guo (2021) indicated that mixed silage overcomes the challenges of alfalfa’s tough silage and tall fescue’s high acidity and low protein, enriching feed with diverse and balanced nutrients. Hou et al. (2015) found that fermentation promoters in grass silage effectively reduce pH and enhance fermentation quality, aligning with this study’s findings.

The NDF content in Group B was significantly lower than that in the control group and Groups D and E (*p* < 0.05), and the ADF content was also significantly lower than that in the control group, Groups C, D, and E (*p* < 0.05). In feed with low NDF content, ruminants have higher consumption and rumination rates. Lai et al. (2014) used inoculants in silage experiments, showing that silage inoculants could reduce the NDF and ADF content in alfalfa silage, which is consistent with the results of this experiment. Other studies have shown that the ratio of concentrate to roughage and the ratio of non-fibrous carbohydrates to neutral detergent fiber (NFC/NDF) are two key indicators for assessing the quality of concentrate feed (Guo et al., 2024). Appropriate ratios of concentrate to roughage and NFC/NDF can improve the production performance of ruminants, maintain rumen health, and increase economic benefits. Moreover, Liu et al. (2024) showed that feeding with 20% NDF level starter feed could achieve better effects, which not only helps optimize the structural morphology of the ileal mucosa but also effectively inhibits intestinal inflammation and helps maintain intestinal health. Lu (2023) showed that feeding finishing pigs with feed containing 16% NDF could enhance the absorption efficiency of fiber by the intestinal flora and promote rapid fat metabolism. In this experiment, compared to the raw material’s NDF and ADF content, all inoculant groups reduced both, with Group B showing a more significant reduction.

Group B’s RFV was significantly higher than Group E’s (*p* < 0.05), likely due to the superior preservation and nutritional value of its silage agent. Group B’s agent had more lactic acid bacteria, boosting lactic acid production, lowering pH, inhibiting harmful bacteria, and reducing toxin residues. Additionally, it may have more microorganisms to degrade plant cell walls, enhancing feed digestibility and nutrient use.

### Effects of inoculants on the fermentation quality of alfalfa silage

Table 5 reveals that lactic acid content increased in all groups compared to the control, with pH values in groups A, C, E, and the control significantly higher than in group B (*p* < 0.05). This may be due to the highly efficient lactic acid production by *Lactobacillus casei* and *Lactobacillus plantarum* in group B, which rapidly ferments carbohydrates, lowers pH, and preserves feed quality. The organic acids from these bacteria inhibit harmful microorganisms, reducing spoilage risk and maintaining feed nutrition. Fermentation by lactic acid bacteria also inhibits yeasts and molds, delaying secondary fermentation and ensuring silage stability.

**Table 5:** Effects of different *Lactobacillus* treatments on fermentation quality of alfalfa silage (% DM).

Item	CK	A	B	C	D	E
pH	5.91 ± 0.04a	5.94 ± 0.08a	5.65 ± 0.02c	5.96 ± 0.06a	5.70 ± 0.03bc	5.75 ± 0.01b
AN/TN(%)	14.30 ± 3.22ab	16.11 ± 0.25a	11.69 ± 0.86b	17.21 ± 2.75a	14.23 ± 1.30ab	15.79 ± 1.91ab
LA(g/kg DM)	5.73 ± 1.46d	12.91 ± 0.29b	17.75 ± 0.52a	10.05 ± 1.28c	8.49 ± 0.89c	12.14 ± 1.35b
AA(g/kg DM)	35.92 ± 1.45b	41.69 ± 2.47ab	29.72 ± 0.44c	47.32 ± 6.27a	37.25 ± 0.89b	38.37 ± 3.94b
PA(g/kg DM)	ND	ND	ND	ND	ND	ND
BA(g/kg DM)	ND	ND	ND	ND	ND	ND

ND, not detected; LA, Lactic acid; AA, Acetic Acid; PA, Propionic Acid; BA, Butyric Acid; AN/TN, Ammonia Nitrogen/Total Nitrogen. The same below. The uppercase letters “A”, “B”, “C”, “D”, “E” and “CK” have been explained as per Table 3. Please refer to the explanations provided in the table. Lowercase letters, “a”, “b”, “c”, etc, indicate significant differences between data. Specifically, data with the same letters in the same column indicate no statistically significant difference, while data with different letters indicate significant differences.

The pH of silage is mainly influenced by lactic acid and the buffering capacity of the forage itself at different cutting stages. Studies by Yun et al. (2017) have shown that inoculants can significantly reduce the pH of alfalfa silage, and cutting stages have a significant effect on pH. inoculants and cutting stages have a significant effect on lactic acid content, and there is a significant interaction effect among them. This study found that all groups maintained a pH above 4.2, likely due to improper inoculant selection or dosage. Alfalfa’s initial moisture, sugar, and buffering capacity affect fermentation, as does the performance of added lactic acid bacteria. Insufficient growth or inhibition of these bacteria in groups A and C, which were inoculated with lactobacillus, resulted in pH levels comparable to the control (CK). The diversity of lactic acid production among lactobacillus species and the role of other microorganisms like yeasts and molds contribute to the complex pH dynamics in silage fermentation (Jia et al., 2018).

Studies by Li et al. (2017) have shown that if the DM content at harvest is below 30%, the yield will be low, the water content will be high, and Clostridium fermentation is likely to occur. In this experiment, the DM content of the Alfalfa raw material was 40.00%, with a lower risk of Clostridium fermentation. The water content of alfalfa at harvest was 60%, and no PA content was detected, which is consistent with their research results. The presence of AA and PA in silage can reduce aerobic spoilage and have a significant negative impact on the feeding preference and intake of ruminants. Alcohols (ethanol and propanol), and esters (ethyl acetate and ethyl lactate) can reduce animal dry matter intake (DMI). AA and PA in silage decrease aerobic decay, significantly harming ruminant feeding preference and intake. Alcohols and esters like ethanol and ethyl acetate can lower animal DMI. Silage contains thousands of metabolites, including VOCs, and future metabolomics studies may uncover more VOCs influencing silage intake (Bandla et al., 2023).

Group B had a lower ammonia nitrogen (AN) content than Groups A and C, with ammonia nitrogen reducing silage quality, primarily caused by clostridia and other bacteria. The higher AN/Total Nitrogen (TN) ratio in Groups A and C may be due to increased clostridia activity. It is necessary to screen for lactic acid bacteria to enhance the quality of alfalfa silage. The inoculant used in Group B improved the quality and reduced the AN/TN ratio, which may be related to the activity and concentration of *Lactobacillus casei* and *Lactobacillus plantarum*.

The silage process can increase the total abundance of ARGs, primarily consisting of tetracycline and fluoroquinolone antibiotics. Efflux pumps are the most important resistance mechanism in alfalfa silage, while sucrose inoculants significantly reduce the total abundance of ARGs and the abundance of tetracycline ARGs (*p* < 0.05). The dominant ARGs in alfalfa silage, including tetracycline (acrB), aminoglycoside (acrD), and aminocoumarin (mdtC), had a significant positive correlation with acetic acid content and the ratio of ammonia nitrogen to total nitrogen (*p* < 0.05). *Pseudomonas aeruginosa* and *stenotrophomonas maltophilia* are the dominant ARG hosts on alfalfa raw material, while *Escherichia coli* is the most important ARG carrier in silage. In summary, sucrose inoculants can reduce the enrichment of ARGs in alfalfa silage (Li et al., 2024).

The LA content in Group B was significantly higher than that in the other groups (*p* < 0.05). LA, as a fermentation product of beneficial silage bacteria, is crucial for successful fermentation and safe storage of silage. Traditionally, high-quality silage should have a lactic acid content between 3.0 and 13% (DM), butyric acid content below 0.2% (DM), and a ratio of ammonia nitrogen to total nitrogen below 10%. In this experiment, the lactic acid content in Group B exceeded 13% at 60 days of silage, and the AN/TN ratio in all treatment groups was greater than 10%. When the ratio of lactic acid to acetic acid is below 3:1, it suggests a lower number of homofermentative lactic acid bacteria, indicating insufficient lactic acid bacteria (Zhang et al., 2005). In the experiment, Group B’s acetic acid concentration rose by 13.84 percentage points over the control, likely due to a higher presence of *Lactobacillus plantarum* and other heterofermentative bacteria, enhancing the silage’s aerobic stability. The lactic acid to acetic acid ratio in all groups was below 3:1, suggesting insufficient lactic acid bacteria for optimal fermentation, favoring heterofermentation. Lactic acid is crucial for lowering silage pH, with pH being a vital indicator of complete anaerobic fermentation.

Lactic acid bacteria are the most critical microbial species in the opening process of silage, and *Lactobacillus brucei* is the most common abnormal fermentation lactic acid bacteria. Yang et al. (2023) demonstrated that the add individually of *Lactobacillus buchneri* or *Lactobacillus plantarum*, or a mixture of *Lactobacillus plantarum* and *Lactobacillus buchneri* in a mass ratio of 1:5, can effectively improve the quality of corn silage. Zhan et al. (2024) further showed that *Lactobacillus paracasei* significantly promotes the fermentation quality of millet silage, with *Lactobacillus buchneri* significantly increasing the rapid degradation of protein fractions and carbohydrates. Reziguli et al. (2023) showing that mixed fermentation agents performed better than single-strain fermentations. However, Chen et al. (2014) found that lactic acid bacteria preparations can enhance the fermentation quality of whole-plant corn TMR, while the addition of propionic acid (PA) can not only partially inhibit lactic acid fermentation but also significantly improve the aerobic stability of fermented TMR. Moreover, Gao et al. (2023) showed that adding cellulase, lactic acid bacteria, formic acid, or other organic acids or enzymes to quinoa silage can improve the silage quality of quinoa. You et al. (2022) found that *Lactobacillus plantarum* PS-8 replicates quickly and stably during the early and middle stages of fermentation, promoting the growth of beneficial lactic acid bacteria and inhibiting the growth of harmful microorganisms, ultimately improving the quality of the silage. Jiang et al. (2023) discovered that the composite silage inoculant consisting of lactic acid bacteria (LAB) and 10% cornmeal, can produce silage with good fermentation quality and nutritional value. In this experiment, compared to the control group, the inoculants in Group B after silage could better improve the quality of alfalfa silage, significantly increasing the LA content and lowering the pH of the silage alfalfa, a result similar to previous conclusions. It is speculated that the reason for this result is that lactic acid bacteria are the most important microbial community for improving silage quality.

Acetic acid is the second most important organic acid after lactic acid (Shao et al., 2022), and it has a unique vinegar-like smell that helps improve the aerobic stability of feed (Peng et al., 2021). Acetic acid plays a crucial role in protein degradation, reducing dry matter loss, and inhibiting the growth and reproduction of harmful microorganisms (Ma et al., 2015). The aerobic deterioration of silage is caused by specific aerobic microorganisms (commonly yeasts) oxidizing the remaining organic acids (Xu and Yu, 2004). If the silage is not sealed promptly after chopping, the excessive fermentation by aerobic microorganisms can increase the sensitivity to aerobic deterioration. Zhang J.-Y. et al. (2022) showed that regardless of whether different types of lactic acid bacteria are used alone or in combination, they can significantly improve the fermentation effect of silage, with *Lactobacillus plantarum* showing the best fermentation effect.

### Comprehensive analysis of different lactic acid bacteria treatments

Using the membership function method, the membership function values of various indicators of alfalfa silage were calculated and then summarized to comprehensively rank the silage quality of various inoculants. The calculation formula is as follows:


$$U\left(X_{j}\right)=\frac{X_{j}-X_{\text{min}}}{X_{\text{max}}-X_{\text{min}}}$$

The pH value, neutral detergent fiber (NDF), acid detergent fiber (ADF), ammonia nitrogen, and acetic acid content are negatively correlated with silage quality. The membership function value calculation formula is as follows:


$$U\left(X_{j}\right)=1-\frac{X_{j}-X_{\text{min}}}{X_{\text{max}}-X_{\text{min}}}$$

Where: U( $X_{j}$ ) represents the membership function value of a certain measured index; $X_{j}$ represents the measured value of that index; $X_{\text{max}}$ represents the maximum value of that index; $X_{\text{min}}$ represents the minimum value of that index. The ranking shows that Group B has the highest membership function value of 0.90 (Table 6).

**Table 6:** Effects of different *Lactobacillus* treatments on nutrient and fermentation quality of alfalfa silage (% DM).

Item	CK	A	B	C	D	E
DM(%FM)	0.35	0.12	1.00	0.00	0.41	0.12
CP(%DM)	0.04	0.31	1	0.26	0.36	0
LA(g/kg DM)	0	0.6	1	0.36	0.23	0.53
WSC(%DM)	0.39	0.25	1	0.14	0	0.68
RFV(%DM)	0.49	0.35	1	0.58	0.34	0
AA(g/kg DM)	0.65	0.32	1	0	0.57	0.51
NDF(%DM)	0	0.67	1	0.65	0.5	0.15
ADF(%DM)	0	0.23	0	0.51	0.51	0.86
pH	0.16	0.06	1	0	0.84	0.68
AN/TN(%)	0.53	0.2	1	0	0.54	0.26
MEAN	0.26	0.31	0.90	0.25	0.43	0.38
RANK	5	4	1	6	2	3

FM, Fresh alfalfa; DM, Dry matter; FM, Fresh matter; CP, Crude protein; Relative Feeding Value, RFV; NDF, Neutral detergent fiber, ADF, Acid detergent fiber; WSC, Water soluble carbohydrate; LA, Lactic acid; AA, Acetic Acid; PA, Propionic Acid; BA, Butyric Acid; AN/TN, Ammonia Nitrogen/Total Nitrogen. The same below. The uppercase letters “A”, “B”, “C”, “D”, “E” and “CK” have been explained as per Table 3. Please refer to the explanations provided in the table.

The inoculant in Group B significantly improved the alfalfa silage quality by reducing pH, NDF, and ADF content, increasing CP and LA content, and effectively inhibiting protein degradation and protease activity, thus lowering the AN/TN ratio. Chen et al. (2023) used membership functions to comprehensively evaluate the whole-plant silage quality of various corn varieties, showing that the membership function values of different varieties and the relative feeding value after silage were very close. Therefore, membership function analysis is accurate in comprehensive evaluation (Wang X.-C. et al., 2024; Wang Y.-P. et al., 2024). Mixing sugarcane stalks with corn stalks at a 3:7 ratio and adding 600 mg/kg cellulase optimizes silage quality, demonstrating cellulase’s fiber-degrading effect and the method’s precision in evaluating silage, reducing bias from single-indicator assessments (Wang et al., 2022).

## Conclusion

The experimental results show that different silage agents have different effects on the fermentation quality and nutritional composition of alfalfa silage. The comprehensive analysis of membership functions indicates that the Xinlaiwang I-alfalfa silage agent can significantly improve the fermentation quality and nutritional value of Alfalfa silage. This research conclusion provides a solid theoretical basis for screening the best lactic acid bacteria inoculants for alfalfa silage fermentation and the development of grass and animal husbandry.

## Data Availability

The original contributions presented in the study are included in the article/supplementary material, further inquiries can be directed to the corresponding authors.
